# Elective amputation and bionic substitution restore functional hand use after critical soft tissue injuries

**DOI:** 10.1038/srep34960

**Published:** 2016-10-10

**Authors:** Oskar C. Aszmann, Ivan Vujaklija, Aidan D. Roche, Stefan Salminger, Malvina Herceg, Agnes Sturma, Laura A. Hruby, Anna Pittermann, Christian Hofer, Sebastian Amsuess, Dario Farina

**Affiliations:** 1 Division of Plastic and Reconstructive Surgery, Department of Surgery, Medical University of Vienna, Währinger Gürtel 18-20, 1090 Vienna, Austria; 2 Christian Doppler Laboratory for Restoration of Extremity Function, Medical University of Vienna, Währinger Gürtel 18-20, 1090 Vienna, Austria; 3 Institute of Neurorehabilitation Systems, Bernstein Focus Neurotechnology Göttingen, University Medical Center Göttingen, Georg-August University, Von-Siebold-Str. 6, 37075 Göttingen, Germany; 4 Department of Physical and Rehabilitation Medicine, Medical University of Vienna, Währinger Gürtel 18-20, 1090 Vienna, Austria; 5 Master Degree Program “Health Assisting Engineering”, University of Applied Sciences FH Campus Wien, Favoritenstraße 226, 1100 Vienna, Austria; 6 Otto Bock Healthcare Products GmbH, Brehmstraße 16, 1110 Vienna, Austria

## Abstract

Critical soft tissue injuries may lead to a non-functional and insensate limb. In these cases standard reconstructive techniques will not suffice to provide a useful outcome, and solutions outside the biological arena must be considered and offered to these patients. We propose a concept which, after all reconstructive options have been exhausted, involves an elective amputation along with a bionic substitution, implementing an actuated prosthetic hand via a structured tech-neuro-rehabilitation program. Here, three patients are presented in whom this concept has been successfully applied after mutilating hand injuries. Clinical tests conducted before, during and after the procedure, evaluating both functional and psychometric parameters, document the benefits of this approach. Additionally, in one of the patients, we show the possibility of implementing a highly functional and natural control of an advanced prosthesis providing both proportional and simultaneous movements of the wrist and hand for completing tasks of daily living with substantially less compensatory movements compared to the traditional systems. It is concluded that the proposed procedure is a viable solution for re-gaining highly functional hand use following critical soft tissue injuries when existing surgical measures fail. Our results are clinically applicable and can be extended to institutions with similar resources.

Hands are essential in interacting with our environment allowing us to perform daily tasks that may put them at risk of injuries. Indeed, hand injuries have been reported to represent almost a third of all work-related injuries, with 30% of them involving either crushing, fracture or amputation^1^. Traumatic events, such as high voltage electrocution, crush, or degloving injuries can be mutilating^2^,^3^,^4^,^5^. After these accidents preservation of life and limb, and restoration of as much of the remaining function as possible are the main surgical priorities^6^,^7^,^8^.

Biological reconstruction will always be attempted first, but in cases of critical tissue loss, functional recovery is not always achieved, resulting in a limb that is not only useless but that also constitutes a painful impediment to the patient^5^,^9^,^10^,^11^,^12^. Similar outcomes can occur in cases where blood supply to the arm is transiently interrupted with ischemia and reperfusion injury, leading to established Volkmann’s contractures^13^. In complicated oncological cases, with loss of entire compartments followed by surgery and irradiation, a permanently useless hand may result^14^. In these dire cases, advanced prosthetic technology may offer the only option to restore a functional hand.

Recently, bionic reconstruction was applied to provide useful hand function in patients with global plexopathies including multiple root avulsion injuries^15^. The patients presented here have suffered critical soft tissue defects and even though their neural pathways remained intact, the remaining muscles in their hand and forearm have been critically damaged limiting their use as control sources for myoelectric prostheses^16^.

Commercially available myoelectric prostheses provide direct control which relies on sequential actuation of up to two degrees of freedom (DoFs) based on muscular contractions that are directly related to prosthetic functions, such as opening or closing the hand. While control of these movements is reliable and robust, the number of DoFs is limited, the actuation sequential, and the control strategy not intuitive for the user. We have thus developed methods, relying on the residual EMG activity, suited for the natural, dexterous control of multiple DoFs in a proportional and simultaneous manner^16^,^17^. In order to maintain robustness of control needed for reliable daily use, while providing high gain in function, we have focused on movements of the wrist and hand.

This study reports for the first time the concept of elective amputation and prosthetic replacement to restore hand function after mutilating injuries. The main challenge addressed by this method is the limited available muscle tissue under voluntary action that can be used for prosthetic control. Here, specific algorithms have been implemented to overcome the problem of limited availability of myoelectric signals needed for dexterous prosthetic control.

## Results

### Standardized Functional Outcome Measurements

**Figure 1:**
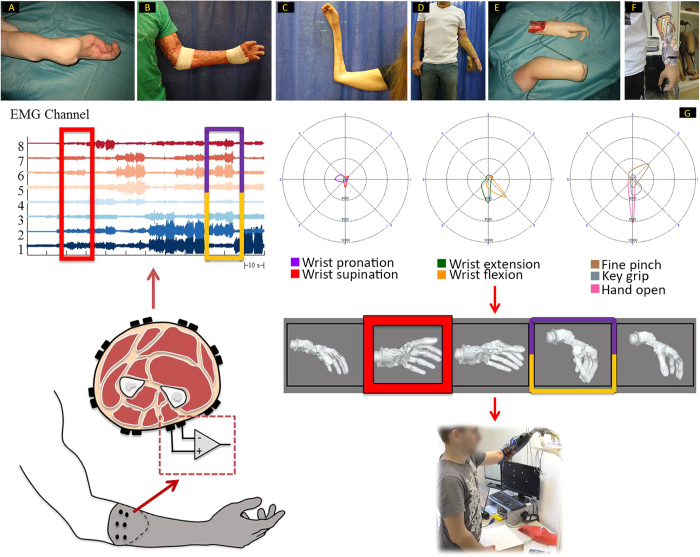
In all cases the reconstructive surgical ladder was attempted first, but with poor functional outcome. The critical soft tissue injuries suffered by the patients in this study were due to (**A**) electrocution, (**B**) degloving injury, and (**C**) complications secondary to compartment syndrome. (**D**) Patient 2 during hybrid hand training. (**E**) Elective amputation of Patient 1. (**F**) Final prosthetic fitting with patient’s 2 own customized socket design and art. (**G**) Schematic of the patient training to achieve proportional and simultaneous control at the level of the wrist. First the patient’s EMG activity is recorded using eight equidistantly placed surface electrodes during a calibration phase. The gross EMG signal is then decomposed into specific patterns that correspond to 7 actions of the prosthetic hand, plus a resting condition. These patterns are uploaded to the prosthetic hand for real time control, which allows for both proportional and simultaneous movements of prosthesis in real-world situations.

In three patients who had suffered critical soft tissue defects (Fig. 1A–C), hand function was measured both before and after the proposed procedure using standardized functional outcome measurements, validated for assessing hand function defects. These included the timed Action Research Arm Test (ARAT), the Southampton Hand Assessment Procedure (SHAP), and the Disability of the Shoulder, Arm, and Hand (DASH) questionnaire^18^,^19^,^20^.

Pre-interventional testing demonstrated dismal sensory and motor hand function in all patients. In fact all patients neglected the use of the impaired hand in daily life, even when bimanual tasks were specifically requested. Post-interventional testing took place at least three months after prosthetic fitting, except in the case of patient 3 who was evaluated 10 days after prosthetic fitting, before she returned to her home country.

Patient 1 (male, 21 at the time of accident) sustained an electrocution injury with both hands severely injured. Both arms and one leg were acutely fasciotomized and multiple surgeries followed to salvage the life and limbs of this patient. On his dominant right hand, the thumb and parts of the middle finger had to be amputated. On the left forearm the entire volar compartment had to be removed and the fifth finger amputated. The soft tissue defect was acutely reconstructed with a large groin flap. After consolidation of all wounds, the right thumb was reconstructed with single homologous finger transplantation, using the fourth finger of his left hand. Six months later, a myocutaneous free flap from his left thigh was transplanted with vascularized fascia lata strips in an attempt to reconstruct finger flexion of his left hand. Additionally, a long vascularized ulnar nerve graft was used to reconstruct median nerve function. Even though all surgeries were successful, the trophic defects of the hand itself were so great that function could not be restored (Fig. 1A). However, residual EMG signals were observable from the volar and the dorsoradial compartment of the forearm. These signals were then assessed for prosthetic control and after much discussion and careful assessment of the patient including psychological evaluation, an elective transradial amputation was performed (Fig. 1E) to allow prosthetic replacement three years after the accident. Patient 1 was fitted with a Michelangelo Hand, Otto Bock Healthcare GmbH. Functional outcomes, as for all the other patients, were measured in three distinct time points: before the procedure took place, during the hybrid fitting where a prosthetic hand was attached to a splint-like device fixed to the patient’s remaining hand (Fig. 1D), and post-intervention following the rehabilitation process (Table 1). The DASH outcome scores for Patient 1 improved from 62 pre to 7.5 after the final prosthetic fitting. ARAT scores gradually improved from 9 to 24 during hybrid fitting, up to 42 upon the procedure. Finally SHAP scores went from 11 pre, to 27 in the hybrid stage and 83 post-intervention.

Patient 2 (male, 28 at the time of accident), suffered a degloving injury of his entire left arm and an avulsion of his right adductor pollicis muscle. After multiple debridements (including muscles, tendons, nerves and both ulnar and radial artery at the forearm), split skin grafts, and negative pressure dressing, over the course of many weeks, the hand was buried under the abdominal skin for soft tissue recovery. Despite these reconstructive efforts the fingers had to be finally amputated at the proximal interphalangeal joints. The patient was discharged from hospital after two months of inpatient treatment. There was still hope of regaining some degree of hand function, but the remaining joints were stiff (see Supplementary Video 1 available) and the quality of his skin grafts continually degraded to open wounds. The hand was completely insensate. In addition, at the elbow and the amputation stumps, the poor skin quality led to recurrent ulcerations and bone protrusions. Unfortunately, attempts at using orthotic supports to encourage remaining hand movement failed due to further skin breakdown (Fig. 1B). After careful assessment and discussion with the patient and prosthetist, the best level of amputation was determined to save remaining forearm and wrist function for optimal prosthetic use. Thus, an elective transmetacarpal amputation was performed three years after the accident. At the same time the chronic ulceration over the left olecranon was debrided and covered with a free vascularized latissimus dorsi flap together with an arthrolysis of wrist and elbow to enhance range of motion of these joints. With respect to the amputation level, the Transcarpal Hand, Otto Bock Healthcare GmbH, was fitted. Similar trends in functional scores as in Patient 1 were observed here (Table 1). Namely, DASH scores improved from 23.33 to 9.17, ARAT outcome values went from 11 before the procedure, to 23 with hybrid fitting up to 36 upon the final fitting and rehabilitation. SHAP values started at 9, only to increase to 32 with hybrid fitting and 70 points upon the procedure.

Patient 3 (female, 20 at the time of accident) fractured both ulna and radial bones of her right forearm. In a follow up appointment later on the same year by the local surgical team, formation of tumors in the upper forearm were noted, with concerns that these might be related to rejection of implanted plates and screws. During the operation to remove these implants, tumor formation was additionally observed in the surrounding muscle, and was later diagnosed as a benign, but aggressive, desmoid tumor. Three operations followed due to residual tumor formation, together with perfusion chemotherapy. The chemotherapy provoked an arterial spasm leading to the development of compartment syndrome of both the volar and dorsal right forearm. Five years after the accident, an attempt to restore some hand function with z-plasties and tendon transfers was not successful. A further fracture of both radius and ulna occurred during aggressive attempts of intraoperative mobilization of the elbow, and was subsequently treated conservatively with a whole arm cast. Due to delayed bone healing the cast was *in situ* for 8 months. After removal, the elbow was stiff and the patient exhibited intrinsic fibrosis of the hand with all fingers positioned in a flexion contracture with no active and very limited passive range of motion. No further help was proposed to the patient at this time. By the time of presentation at our center, the hand was completely stiff, atrophied and with no useful sensation (Fig. 1C). However, some faint activity in her flexor and extensor muscles in the upper forearm could be detected. The patient had previously been offered an above elbow arm transplantation by a different surgical team, but declined in favor of the procedure proposed here, with the main motivation of being of child bearing age without completed family planning and fear of the effects of immunosuppression. After assessment, discussion, intensive EMG signal training and hybrid fitting, an arthrolysis tendon lengthening and soft tissue augmentation at the elbow was performed to allow adequate range of motion. An elective transradial amputation was then done to allow fitting of the Myobock Hand, Otto Bock Healthcare GmbH. Even though the post-procedure outcome measurements took place before the full rehabilitation process was conducted, only 10 days after the prosthetic fitting, improvements with respect to the initial outcome values were observed across all tests (Table 1). DASH scores improved from 35.83 to 26.67. ARAT score started at 3 before, then went to 16 with the hybrid setup, and reached 30 at final evaluation. Lastly, SHAP score improved from 9 to 27 with hybrid fitting and remained at 27 just after the fitting before the end of the full rehabilitation process.

All functional outcomes are documented in Table 1 and demonstrated in Supplementary Video 1. Across all patients, the DASH outcome scores improved from a mean of 40.4 ± 19.7 to 14.5 ± 10.6, the mean ARAT score also improved from 7.67 ± 4.16 during intermediary testing with a hybrid hand to 21.00 ± 4.36, and, after final prosthetic fitting, to 36.00 ± 6.00. This trend of gradual improvement was also observed during SHAP testing. Before the intervention, the patients scored a mean of 12.00 ± 3.61, with hybrid fitting 28.70 ± 2.89, and, after final fitting, 60.00 ± 29.30.

### Assessment of Simultaneous, Proportional & Dexterous Control

**Figure 2:**
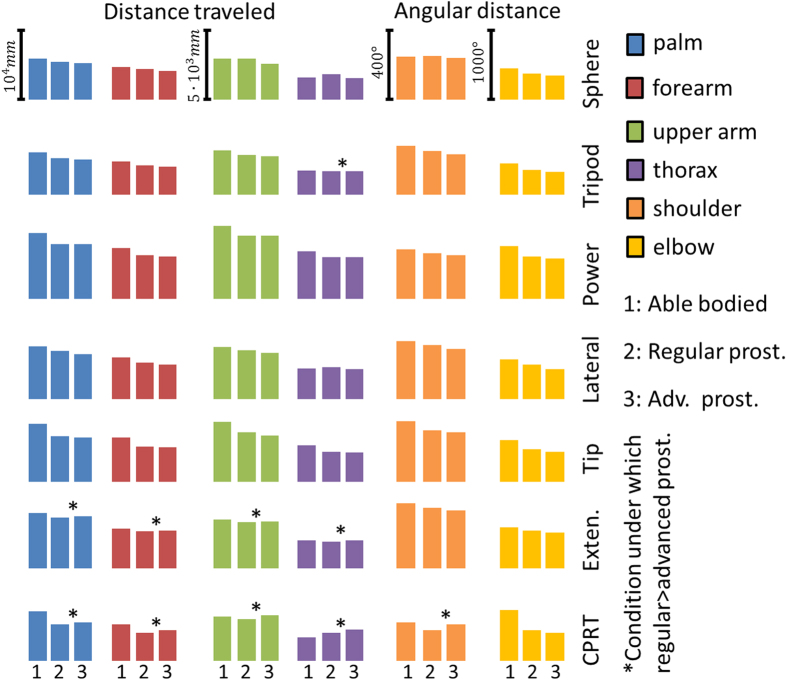
Recorded kinematics with respect to anatomical segments and joints across different sub-groups of SHAP test and CPRT for able-bodied group (1), Patient 1 with classical prosthesis (2) and Patient 1 with advanced prosthesis (3). Notably, in terms of kinematics, Patient 1 was more efficient during the execution of tasks than on average all five able-bodied participants by requiring less overall motions during the execution of tasks.

**Figure 3:**
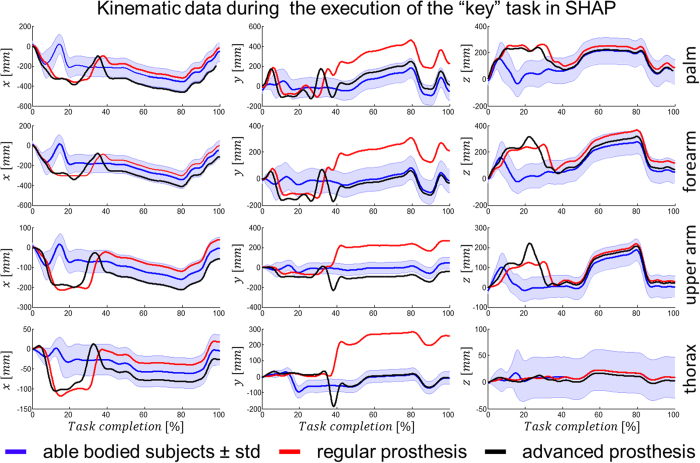
Recorded centroid traces of respective anatomical sections across all three axes during the execution of an example task of the SHAP test, the key task, for able bodied group, Patient 1 with classical prosthesis and Patient 1 with advanced prosthesis. The origin of the coordinate system for the each anatomical section was set at the starting (resting) point of the respective section with z axis pointing vertically upwards, parallel to the patient’s vertical axis and x, y axes aligning with the transverse and the sagittal one.

After careful examination of his muscular activity, patient 1 was fitted with a more advanced control system in addition to his standard prosthesis which he has been using for 18 months. At that time, no major problems with system fitting or running had been observed. The advanced custom fitted system consisted of a Michaelangelo prosthetic hand that included wrist flexion/extension and rotation and two hand grasps, palmar and lateral. The whole system was intended strictly for laboratory investigations and not for home use at this time. A short training and familiarisation with the implemented control algorithm^17^ which allowed the control of these additional DoFs using both proportional and simultaneous movements at the wrist (Fig. 1G) was made. Patient 1 was asked to perform SHAP and the clothespin relocation test (CPRT) using both the advanced and his regular prosthetic. Kinematics of his upper limb and trunk were recorded using a motion capture system during prosthesis manipulation and compared between prostheses. The data sets were evaluated against five able-bodied controls using their non-dominant natural hand as a reference. The advanced prostheses could be controlled by the patients with a similar SHAP score (68) as the regular prosthesis. However, the kinematics analysis during the tests indicated that the overall movements were closer to the natural movements of the able-bodied subjects with the advanced system that allowed more natural-like hand use. These outcomes were observed despite the fact that the patient had substantially less training with the advanced system than with the regular prosthesis (Fig. 2, Supplementary Video 2). The functional advantages of the advanced system were particularly evident for tasks that required coordinated movements of the wrist, such as using a key or moving clothespins from a horizontal to a vertical bar (Fig. 3, Supplementary Video 2).

### Pain & Quality of Life

Pain scores relevant to the affected limb were evaluated using a 10-point visual analogue scale (VAS)^21^. Prior to the procedure, the pain scores were 0, 0.5, and 1.5 for patients 1, 2, and 3, respectively. All three patients reported no pain after the procedure.

All patients were tested for changes of quality of life after the procedure as measured by the SF-36 Health Survey (4-week recall)^22^. Assessed sub-items and summary scales are documented in Table 1. Following final prosthetic fitting and regained ability to use both hands, a marked improvement of physical functioning was noted in all patients. Overall, bodily pain was reduced successfully for all three patients, exhibiting optimized social and emotional role functioning. Additionally, mental health was enhanced. General health perception remained the same in Patient 3 and improved in Patient 1 and 2, implying the importance of functional recovery in these patients.

In addition to the standardized questionnaires showing improvement in quality of life, the patients reported improvement of social engagement and general activities of daily living. Interaction with their environment was simplified and physical appearance with regards to self-confidence was also enhanced. After prosthetic replacement, Patient 2 was able to continue working as a manual labourer and Patient 1 returned to work as an electrician after receiving the treatment.

## Discussion

In the three patients presented in this study, the procedure combining surgical reconstruction, tech-neuro-rehabilitation program, sophisticated signal extraction, carefully planned elective amputation, and implementation of advanced prosthetic systems enabled hand function where biological reconstruction had previously failed.

Functional outcomes following mutilating hand injuries are dependent on several factors which can influence the resulting hand use^23^. In particular, the worst functional outcomes are associated with multiple levels of injury, crush with subsequent tissue ischemia, or massive loss of functional tissue^24^. The patients in this study suffered such devastating injuries, either due to electrocution, degloving or compartment syndrome. All extended far beyond the hand, affecting the limb at multiple levels, and are as such representative of the difficult cases that confront reconstructive surgical teams. As exemplified in this study, all biological reconstructive means were first attempted to restore function, yet the functional outcomes were poor, even years after the accidents. These patients may be considered for hand transplantation, but evidence suggests that prosthetic reconstruction provides equivalent motor function without the risk of life-long immunosuppression and lengthy rehabilitation^25^. As there were no other autologous means that could be attempted in these patients, bionic substitution following elective amputation was offered to them as the last resort to gain hand function. This report demonstrates that recovery of hand function in patients with critical soft tissue injuries is achievable with this multidisciplinary reconstructive approach.

The interface for prosthetic control in this study was established by muscle signals. Muscles act as translators of intuitive neural information and biological amplifiers of nerve signals. The requirements for successful prosthetic control are related to consistency, accuracy, intuitiveness, function, and, above all, robustness over a broad range of conditions, including activities of daily living. Despite important developments in brain and nerve interfacing^26^,^27^, muscle interfacing is the only current viable way for robust use of prosthetic systems. This makes critical soft tissue defects a particular challenge, as the muscles have been destroyed in the original injury, ischemia, surgical debridement or irradiation and, therefore, myoelectric signal recording is challenging. The technique we have developed identifies and trains the gross EMG signal for myoelectric control, without the need for implanting electrodes or other interfaces^28^.

The myoelectric control methods implemented in this study are commercially available devices that have been customized to each patient’s requirements. In addition to classic direct control algorithms, in order to further explore the possibilities of the presented approach, an advanced control algorithm, previously tested in realistic conditions on transradial amputees^29^, has been adapted for use in one of the patients presented in this study. With this algorithm, the main advantage over the industrial state of the art in myocontrol is the possibility of controlling multiple DoFs concurrently, which corresponds to natural movements, in an intuitive way. We demonstrated that even patients with faint myosignals, as presented here, are able to control 3.5 DoFs in a dexterous way. This allowed execution of everyday tasks such as picking up coins, pouring a glass of water or using a key in a more natural way for the patient, avoiding unnatural gross movements at the elbow and shoulder. Even in conditions for which less DoFs were sufficient for completing the tests, the more advanced control system in a combination with the highly actuated prosthesis resulted in less accessory movements, which implies that the tasks were executed more naturally.

The results of this study have been presented for situations closely resembling daily activities, with customized sockets mimicking those used by the patients at home and an array of tests that quantified in depth their functional capacity. Moreover, all patients used the prosthetic systems they received in this study at home and during work. This way of assessing active prosthesis control provides direct evidence of the real functional gain achieved by the proposed method, and is the direct basis for translating research outcomes into clinical systems for daily use. Indeed, the three patients currently use their prostheses on a daily basis for an average of 6 to 14 hours per day.

In addition to severe motor impairment, major upper limb injuries are not only physically devastating, but also contribute greatly to psychological harm, leading to such comorbidities as anxiety and depression^30^,^31^. If biological reconstruction fails, as in the three patients described in the present study, the patients are confronted with the prospect of life with a useless and esthetically inferior limb. This not only impacts their ability to conduct normal tasks of daily living, but also affects their professional and personal development with negative repercussions on self-image and self-worth^32^. Addressing the psychological impact of mutilating hand injuries is likely to improve functional outcomes^33^. In relation to failure of limb salvage, amputation has been shown to have no disadvantage from a psychological perspective in critical soft tissue defects^34^, and wearing prosthesis helps amputees to maintain a body image in which the missing limb is matched to the prosthesis^35^. As such, replacing a non-functioning limb with a prosthetic limb does not only improve the patient’s functional capacity, but may also be psychologically rewarding.

It should be noted that, even though the procedure itself does not contain any specific risks inherent to this type of surgery, standard concerns as in any similar intervention do apply. Concerns are related to not achieving the expected improvement of functionality, ending up with an irreversible loss of a hand, having the second thoughts once the amputation has been performed, or worst of all if the amputation stump is painful. Therefore the steps that precede the amputation are of utmost importance and consist of detailed psychological evaluation during decision making process, hybrid fitting with in-depth hand, and prosthesis function assessment, including sensitivity tests. During all these steps contraindications, which include unrealistic expectations to prosthetic device performance, negative hybrid hand outcome due to lack of motor function and useful sensitivity of patient’s affected limb are clear indicators for patient’s exclusion from the procedure.

Once the standard reconstruction techniques are not able to provide satisfactory outcomes, as in cases shown here, alternative options need to be considered. As has been demonstrated recently, the careful application of bionic reconstruction can restore hand function in neurological injuries^15^. The same philosophy was applied in this study inthree patients with critical soft tissue injuries, with entirely different challenges. The surgical procedures and rehabilitation program were similar to those used for neurological injuries, albeit for different indications, but with similar successful results in terms of recovered function. Moreover, intact efferent pathways allowed the demonstration of highly dexterous control (3.5 DoF simultaneously and proportionally controlled) in one of the patients treated. The technique elaborated here is a clinical reality and not a laboratory-based concept, and institutions with similar resources and skill could apply this method to patients with similar devastating injuries.

## Methods

Three patients, who had suffered critical bone and soft tissue defects, gave informed consent to take part in this study between January 2010 and November 2014. The ethical approval was obtained from the Medical University of Vienna’s Institutional Human-Studies Review Board (Ethic Commission Number 1209/2012). The methods were carried out in accordance with the relevant guidelines.

### Clinical Evaluation

Initial suitability was assessed in the specialist hand clinic, including neurological and musculoskeletal examination of upper limb function. Patients also underwent high-resolution ultrasound and nerve conduction studies to assess the state of existing muscles and nerves. Other factors that were assessed included quality of life metrics (Short-Form 36) and associated pain scores^22^. All patients underwent psychological evaluation performed by a qualified psychologist both before and after the intervention. The evaluation contained a structured clinical interview (SCID) and Freiburger assessment to evaluate adequate coping strategies (FKV-LIS) and behavioral observations^36^.

### Tech-Neuro-Rehabilitation Program

After surgical reconstruction (Fig. 1A–C), and before amputation (Fig. 1E), cognitive training was initiated through rehabilitation using visual feedback. First, the generation of consistent muscle electrical activity was trained using surface EMG biofeedback. While the non-functioning hand was in place, the use of virtual rehabilitation encouraged the patients by demonstrating that they still could control hand’s function. Repeated surface EMG recordings showed that this training improved signal quality and control. The patients could then practice the different functions of the prosthesis through virtual rehabilitation and table-top prosthesis before the actual fitting. This process took between a few days and several weeks depending on the cognitive training requirements of the patient and the time elapsed since injury. Once confident in this setting, the patients were fitted with a “hybrid hand” (Fig. 1D). The device provided direct proof for the patients that they could achieve better hand function using the prosthesis than their non-functioning biological hand (see Supplementary Video 1 available). Depending on their outcome with the virtual and hybrid prosthetic systems, the patients were offered different control algorithms and were tested with them.

### Control Algorithms

Based on the training phase, it was decided for patients 2 and 3 to use conventional direct control. They could articulate at least one single DoF and the speed of the prosthetic was linearly correlated to the level of activity of the dedicated agonist or antagonist muscle depending on the direction within the DoF. Patient 3 was fitted with the rotation unit, in addition to hand opening/closing, and therefore was given an opportunity to switch between DoFs using co-contraction of agonist/antagonist with classic control. Moreover, Patient 1 presented sufficient signal quality to attempt advanced simultaneous and proportional control of the multifunctional prosthesis, with the possibility of acting intuitively on 3.5 DoFs^17^. The tests on this patient are reported both for classic control algorithms (direct control) as well as the more advanced algorithms. The classic control algorithms were those for which the patient was first trained and which he used at home.

### Functional Assessment

The evaluation of regained function was done with a number of established tests for hand function, including tests that resemble activities of daily living, both using the hybrid system with the prosthesis mounted in parallel to the non-functioning hand and after elective amputation and prosthetic fitting. The global upper extremity function was evaluated both pre- and post-intervention with the ARAT, SHAP, and the DASH questionnaire, which monitor hand and upper extremity function closely related to activities of daily living^18^,^19^,^20^. The ARAT has been validated for use with patients with cognitive impairment of hand control, the SHAP for the assessment of pathological and prosthetic hand function, and the DASH for patients with musculoskeletal diseases and injuries to the upper limb^18^,^19^,^20^.

The ARAT consists of four sections with different tasks, a maximum of 57 points attainable and takes about 10 minutes. The SHAP consists of eight light and eight heavy abstract objects, and 14 Activities of Daily Living (ADL) with each task timed by the patients themselves^18^. Normal hand function is regarded as equal to 100 points in the SHAP. Depending on the dexterity of the patient it needs about 30 minutes time. Bimanual tasks and impariment in daily life were rated with the DASH, where a score of 100 indicates the worst and 0 indicates the best hand function^20^. The DASH was not performed using the hybrid device, as it was a training device not available at the patients’ homes (a requirement of the DASH questionnaire).

### Motion Capture for Assessment of Dexterous Advanced Control

In addition to the tests performed by the other patients, Patient 1 completed the CPRT. The CPRT is an adapted clinical test from the Royal Graded Pinch Exerciser^37^ that involved transferring clothespins of various strengths from a horizontal bar to a vertical one. This test is particularly useful to assess rotation, flexion and extension movements at the wrist. All tests were performed with both the traditional fitting and the advanced control prosthesis.

In order to accurately analyze the kinematics of the upper limb and trunk during prosthesis usage, 17 passive reflective markers were secured to the dorsum of the participant’s affected arm at well-defined anatomical positions to clearly identify all movements made by the arm. Reference markers were positioned above C7, sternal angle, and the acromion processes. Clusters of markers were used to define the centroids of four segments of interest – palm, lower arm, upper arm and thorax. An eight-camera VICON MX+ optoelectronic motion capture system (Oxford Metrics Ltd., Oxford, UK) was used to capture the movements of the passive markers at a sampling rate of 200 Hz. The coordinates of the markers were recorded in 3D-space in relation to time. The motion data were processed using MATLAB 2013a (Mathworks, Massachusetts, USA).

For assessing the movements of the upper limb in relation to natural movements, five able-bodied subjects were also measured as an age-matched normative group (22–35 years, all male and right-side dominant). They were instructed to perform both the SHAP and the CPRT using their non-dominant hand while their motion was recorded with the reflective marker montage as described above. In this way a reference data set was obtained.

### Pain and Quality of Life Assessment

Pain scores were evaluated using visual analogue scales (VAS). All study patients were tested for changes of quality of life after the procedure as measured by the SF-36 Health Survey (German Version, 4-week recall)^22^. The questionnaire addresses 8 independent subscales: physical functioning, physical role functioning, bodily pain, general health, vitality, social role functioning, emotional role functioning, and mental health. Each listed subscale ranges from 0 (= minimum) to 100 (= maximum). This scale also includes bodily pain as a sub-item with a higher score indicating less pain. Based on the subscales, two superior physical and mental component summary scales can be identified. These have mean values of 50 and a standard deviation of 10. For example, a patient with a psychological sum scale of 65 exhibits above average mental health compared against published age- and sex-matched norm samples of an overall representation for the German population. The study patients completed the questionnaire prior to the procedure and after the prosthetic device had been incorporated into the user’s activities of daily living.

## Additional Information

**How to cite this article**: Aszmann, O. C. *et al.* Elective amputation and bionic substitution restore functional hand use after critical soft tissue injuries. *Sci. Rep.* **6**, 34960; doi: 10.1038/srep34960 (2016).

## Supplementary Material

Supplementary Information

Supplementary Video 1

Supplementary Video 2

## Figures and Tables

**Table 1:** Functional outcome scores and quality of life assessment for all patients pre- and post-procedure.

Functional outcome scores	Patient 1			Patient 2			Patient 3		
	Pre	Hybrid	Post	Pre	Hybrid	Post	Pre	Hybrid	Post
DASH	62.00	/	7.50	23.33	/	9.17	35.83	/	26.67*
ARAT	9	24	42	11	23	36	3	16	30*
SHAP	11	27	83	16	32	70	9	27	27*
**Quality of life scores (SF-36)**	**Pre**	**Post**		**Pre**	**Post**		**Pre**	**Post**	
Physical functioning	75	95		70	95		85	90	
Physical role functioning	100	100		0	25		100	100	
Bodily pain	84	100		84	100		84	74	
General health perception	87	100		72	82		72	67	
Vitality	60	50		75	70		80	90	
Social role functioning	100	100		87.50	100		100	87.50	
Emotional role functioning	100	100		33.30	66.70		100	100	
Mental health	84	88		68	72		84	96	
Physical comp. sum. scale	50.90	57.70		43.80	51.90		51.20	48.80	
Mental comp. sum. scale	56.60	53.80		48.00	50.20		58.00	60.80	

Notes: DASH - Lower score represents better function. In both the ARAT & SHAP higher score represents better function. Normal hand function is regarded as equal to or above 100 points in the SHAP. (*) Patient 3 was evaluated at 10 days after prosthetic fitting, and as lives in a separate country was unavailable for further follow up by our group. SF-36 ranges from 0 representing the poorest quality of life, and 100 as the best (100 in the sub-item bodily pain indicates a pain-free state).
