# Supplementary material for: Elective amputation and bionic substitution restore functional hand use after critical soft tissue injuries
Source: Sci Rep. 2016 Oct 10;6:34960. doi: 10.1038/srep34960 (PMC5056343; doi:10.1038/srep34960)
Supplement: Supplementary Information [file srep34960-s1.pdf]

# Elective amputation and bionic substitution restore functional hand use after critical soft tissue injuries

Oskar C. Aszmann[a,b,*], Ivan Vujaklija[c], Aidan D. Roche[b], Stefan Salminger[a,b], Malvina Herceg[f], Agnes Sturma[b,d], Laura A. Hruby[b], Anna Pittermann[a], Christian Hofer[e], Sebastian Amsuess[e] & Dario Farina[c]

[a]Division of Plastic and Reconstructive Surgery, Department of Surgery, Medical University of Vienna, Währinger Gürtel 18-20, 1090 Vienna, AUSTRIA
[b]Christian Doppler Laboratory for Restoration of Extremity Function, Medical University of Vienna, Währinger Gürtel 18-20, 1090 Vienna, AUSTRIA
[c]Institute of Neurorehabilitation Systems, Bernstein Focus Neurotechnology Göttingen, University Medical Center Göttingen, Georg-August University, Von-Siebold-Str. 6, 37075 Göttingen, GERMANY
[d]Master Degree Program "Health Assisting Engineering", University of Applied Sciences FH Campus Wien, Favoritenstraße 226, 1100 Vienna, AUSTRIA
[e]Otto Bock Healthcare Products GmbH, Brehmstraße 16, 1110 Vienna, AUSTRIA
[f]Department of Physical and Rehabilitation Medicine, Medical University of Vienna, Währinger Gürtel 18-20, 1090 Vienna, AUSTRIA

## Supplementary Information

**Supplementary Video 1 Functional outcomes observed in Patient 2 pre- and post- intervention as well as during the hybrid fitting**

**Supplementary Video 2 Post-intervention follow up of Patient 1 fitted with the advanced prosthetic system using simultaneous and proportional control**
